# Enhancing independent eating among older adults with dementia: a scoping review of the state of the conceptual and research literature

**DOI:** 10.1186/s12912-020-00425-x

**Published:** 2020-04-21

**Authors:** Alvisa Palese, Valentina Bressan, Mark Hayter, Roger Watson

**Affiliations:** 1 Department of Medical Sciences, University of Udine, Viale Ungheria, 20, 33100 Udine, Italy; 2 Faculty of Health Sciences, University of Hull, Hull, UK

**Keywords:** Eating difficulties, Mealtime difficulties, Eating performance, Dementia, Eating intervention, Nursing homes

## Abstract

**Background:**

Addressing eating difficulties among older individuals with dementia living in nursing homes requires evidence-based interventions. However, to date, there is limited evidence of effective interventions designed to maintain and/or increase independent eating. In a field in which evidence is still lacking, a critical analysis of the state of research describing its main features can help identify methodological gaps that future studies should address. Hence, the aim of this study was to map the state of the research designed to maintain and/or promote independent eating in older individuals with dementia living in nursing homes.

**Methods:**

A scoping review was performed by following the Preferred Reporting Items for Systematic Reviews and Meta-analyses. Reviews and conceptual analyses performed with different methodological approaches, published in indexed journals, and written in English were included. Keywords Were searched for in the MEDLINE, the Cumulative Index of Nursing and Allied Health, and in the Scopus databases to identify papers published up to 31 May 2018.

**Results:**

17 reviews were included, assessing interventions’ effectiveness (*n* = 15) and providing conceptual frameworks for eating/mealtime difficulties (*n* = 2). Conceptual frameworks supporting interventions’ effectiveness have rarely been described in available studies. Moreover, interventions tested have been categorized according to non-homogeneous frameworks. Their effectiveness has been measured against (1) eating performance, (2) clinical outcomes, and (3) adverse event occurrence.

**Conclusion:**

An increased use of conceptual frameworks in studies, as well as greater clarity in intervention categorization and outcomes, is necessary to enhance the reviews’ value in providing useful cumulative knowledge in this field. Interventions delivered should embody different components that integrate individual, social, cultural, and environmental factors, while when evaluating an intervention’s effectiveness, eating performance, clinical outcomes and adverse events should be considered. Together with more robust studies, involving clinicians could prove to be useful, as their knowledge of practice developed from direct experience can help develop innovative research questions.

## Background

The increased need for care because of functional dependence in activities of daily living among older individuals with dementia has been documented as the strongest predictor of nursing home (NH) admission [1, 2].

Several studies have evaluated the effectiveness of interventions that promote independent toileting, transferring, and locomotion (e.g., de Souto Barreto et al. [3]). Similarly, studies designed to determine ways to optimize independent eating and to maintain it as long as possible have been performed to date. However, because of the paucity of available evidence, interventions to promote and maintain independent eating are still among the top priorities in the NH agenda [4]. In fact, as recently documented, around one-third of NH residents’ [1] cognitive and functional abilities decline, becoming dependent on others for eating.

Despite the daily efforts of healthcare professionals (HCPs) and family caregivers to promote independence in eating and in providing adequate support, in the long run unintentional weight loss, malnutrition, dehydration, pneumonia, decreased quality of life, and in some cases death [1] have been documented among older individuals with dementia living in a NH. Moreover, eating dependence has been shown to raise important ethical issues (e.g., when the resident refuses to eat) and to affect the residents’, HCPs’ (e.g., nurses, nurses’ assistants), and/or family caregivers’ quality of life [4].

Various definitions have been developed in the literature in this field. “Feeding” or “Eating” was first defined as the act of moving food from a plate to the mouth and swallowing it [5]. The term “eating difficulty” was established to describe any aspect in the process that might lead to reduced food and liquid intake [6]. Later, the concept of “mealtime difficulties” was developed as an overarching term that referred theoretically to aversive eating, feeding, and meal behaviours, and implied interpersonal, sociocultural, and environmental factors [7]. More recently, the concept of “eating performance” has been introduced to describe the functional ability of putting food into the mouth [8]. However, none of these keywords is included yet in the Medical Subject Heading dictionary of Medline databases, making it difficult both for clinicians and researchers to consult the available evidence.

Eating difficulties are a daily concern for both HCPs and family caregivers [9]. During the early stages of dementia, older individuals require minimal support, in the form of prompts or advices. In advanced stages, promoting eating independence requires the resident’s participation in maintaining attention, in opening the mouth, and in swallowing while helped in eating. It also requires adequate time for care and an environment without distractions [10]. Above all, it requires positive attitudes from the HCPs or family caregivers [11] and a proper relationship with residents with dementia [10].

Promoting independence in NHs is even more complex, as HCPs must assist many residents with different degrees of eating difficulties. However, despite a large number of studies available as documented in available reviews [10], at this stage of research in the field, no intervention can be recommended as gold standard [12]. In a field in which evidence is still lacking, a critical analysis of the conceptual and research literature describing its main features can help in identifying methodological gaps that future studies should address.

Therefore, the role of this scoping review is to offer an overview of aims, conceptual frameworks, interventions, and outcomes studied to date in the field of eating difficulties in older individuals with dementia living in NHs.

## Methods

### Study design and methodology

A scoping review was performed [13–15] following the framework developed by Arksey and O’Malley [13] and recently revised [16, 17]. The scoping review methodology was chosen due to its capacity to support a knowledge synthesis addressing an exploratory research question and allowing to map conceptual framework, different types of evidence, and gaps in a research field area [16]. For the present scoping review the following steps were performed: 1) research question formulation; 2) identification of relevant studies; 3) selection of relevant studies; 4) data charting, and 5) collection, summary, and report of findings [16, 17]. Results were reported following the Preferred Reporting Items for Systematic Reviews and Meta-analyses (PRISMA-ScR) extension for such reviews [18] as reported in Supplementary table 1.

### Research question

The following research question was formulated: What aims, conceptual frameworks, interventions, and outcomes designed to improve eating independence among older individuals with dementia living in NHs have been studied to date?

### Relevant studies and rationale

Based on the research question, the following inclusion criteria were established:

1. Secondary studies (e.g., meta-analyses, systematic reviews, overviews, narrative reviews, integrative reviews) that summarized the state of art of research in this field and its gaps, and provide future study directions, were included;
2. Concept analyses used to summarize knowledge based on the review of the literature: these were included to ensure a comprehensive map of the conceptual frameworks available in this research field, and
3. Papers written in English and published in journals indexed in the Medline, CINAHL, and Scopus databases, up to 31st May, 2018.

Therefore, the following were excluded: 1) case reports, editorials, letters, and expert opinions; 2) primary quantitative and qualitative studies; 3) instrument validation studies; 4) reviews focused on the effects of nutritional interventions (e.g., providing high calorie food; food enriched with protein or nutrients to improve body mass index or weight) or on enteral nutrition as an intervention, and 5) grey literature.

**Fig. 1:**
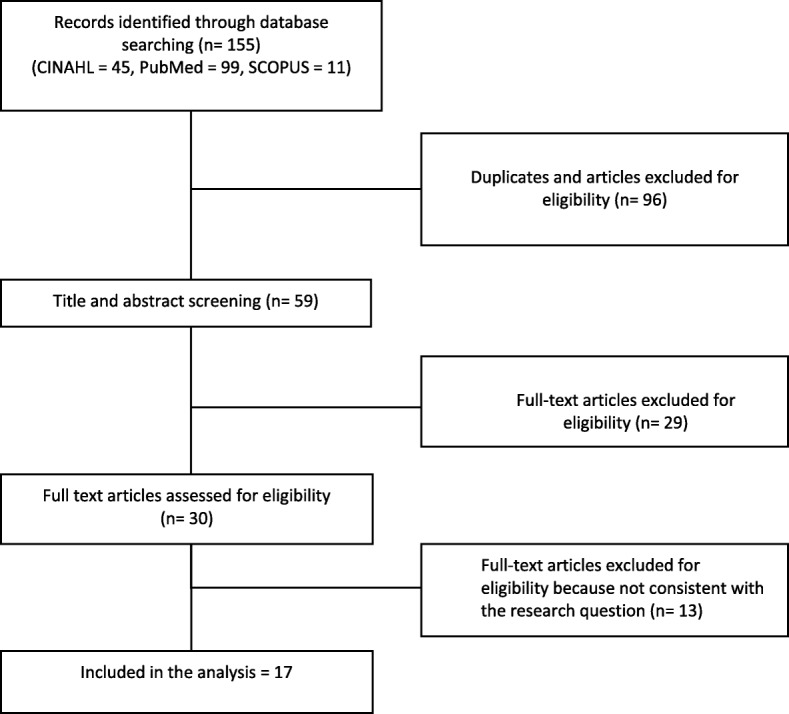
The process of study inclusion: flow diagram

The following terms were used associated with the Boolean operator ‘AND/OR’: *“eating difficulties,” “feeding difficulties,” “mealtime difficulties,” “eating performance,”, “eating interventions”, “feeding intervention”, “dementia.”* Studies emerged were screened independently by two reviewers who analysed their titles and abstracts against the inclusion criteria. Thereafter, the full texts of the eligible reviews were retrieved and reviewed independently by the same two researchers. A list of the references cited in the reviews included was examined, and their citations, as documented in the Scopus database, were also checked. Decisions about the final inclusion of 17 reviews were based on reading the full text and on the agreement between researchers. In Fig. 1, the process of study inclusion has been reported.

### Data extraction

The reviews and the concept analyses included were read carefully. Then, the following data was extracted: (a) aims(s); (b) study design (e.g., systematic review); (c) target population; (d) conceptual framework(s), if any, used to design/explain the intervention’s effectiveness in the primary study included in the review; (e) intervention(s) tested to promote eating independence and their categorization as provided in the review, and (f) outcomes measured as summarized by the reviews included. This data was extracted and recorded independently by two researchers in an ad hoc grid and then agreed upon. The grid was piloted on a preliminary fashion in one review in order to check its consistency and feasibility.

### Data analysis, collation, and summary

The aim(s), target population, as well as settings described in the reviews included were extracted and recorded in the grid. The conceptual frameworks used to justify the intervention’s effectiveness were also scrutinized and extracted when reported explicitly in the reviews [19]. When not reported explicitly, researchers used an inductive process to identify the conceptual frameworks underlying the intervention(s) the reviews reported, and categorized them through content analysis [20] performed by two researchers, independently and then agreed upon. Conceptual frameworks emerged were based on: biological, cognitive, emotional, and behavioural processes, and individual, interpersonal, and environmental processes. Each conceptual framework was described briefly by also searching and identifying eminent authors in the field. Moreover, to validate the inductive categorization process [20], examples of interventions documented in the reviews included were reported briefly.

The interventions were extracted as categorized by the author(s) of each review. Then, given the differences in categorization used across reviews, interventions were categorized according to the literature available in the field [21–23] as follows: (1) environmental interventions, including, for example, changes in routine, context, and environments, and (2) behavioural interventions, including educating or training individuals with dementia or their caregivers and relevant others.

Finally, outcomes documented in the included reviews were analysed and categorized in three main dimensions based on content analysis [20]: (a) performance in eating considered as the functional activities of getting food and drink into the mouth, (b) clinical effects (e.g., increased weight), and (c) adverse events or negative outcomes (e.g., pneumonia [6]) as a consequence of the interventions evaluated for their effectiveness.

## Results

### The aims of the included studies

As shown in Table 1, a total of 17 reviews published between 1993 and 2016 were included. These focused-on interventions’ effectiveness (*n* = 15) or were concept analyses of feeding and/or mealtimes difficulties (*n* = 2). The amount of primary studies included in the emerged reviews was 278; of these, a total of 64 studies were analysed in at least two reviews.

**Table 1:** Studies included in the scoping review

Focus^**a**^	Author(s), years, Country	Main purposes	Study design
Intervention(s) effectiveness	Watson, 1993 (UK) [6]	Issues in measuring feeding problems; direct and indirect interventions; measuring intervention effectiveness *Target population*: older adults with dementia in any context	O + research agenda
	Amella, 1998 (USA) [24]	Direct interventions (dietary needs) and indirect interventions (social, cultural, and interactive components of mealtime) *Target population*: elderly individuals; special strategies for people with cognitive disabilities	O + clinical protocol
	Manthorpe & Watson, 2003 (UK) [25]	A census of areas well-developed on feeding difficulties, as well as of areas with little knowledge and potential improvement *Target population*: patients with dementia and their caregivers in any setting	Position paper + research agenda
	Watson & Green, 2006 (UK) [26]	Interventions to promote oral nutritional intake *Target population*: older people with dementia in any setting	SR
	Aselage et al., 2011 (USA) [7]	Exploration of the state of the science related to assisted hand-feeding *Target population*: people with dementia in NHs	O
	Chang & Roberts, 2011 (USA) [27]	Areas of feeding difficulties (initiating feeding, maintaining attention, getting food into the mouth, chewing food and swallowing food); their specific manifestations, observable behaviour associated with each; multidisciplinary and feeding strategies documented as effective *Target population*: patients with dementia in Hospitals and NHs	O
	Hanson et al., 2011 (USA) [28]	Benefits of oral feeding options *Target population*: people with dementia in LTC	SR
	Jackson et al., 2011 (UK) [29]	Effectiveness of mealtime interventions *Target population*: adults over the age of sixty with dementia living in any setting (no home)	SR
	Abbot et al., 2013 (UK) [21]	Effectiveness of mealtime interventions *Target population*: elderly individuals living in residential care, including also people with dementia	SR + MA
	Liu et al., 2014 (USA) [10]	Effectiveness of interventions on mealtime difficulties *Target population*: older people with dementia in any setting	SR
	Bunn et al., 2015 (UK) [30]	Effectiveness of interventions to increase fluid intake and reduce risk of dehydration *Target population*: older adults who could drink living in residential, LTC special dementia units	SR
	Douglas & Lawrence, 2015 (USA) [31]	Evaluate the research on environment-based interventions to improve nutritional status *Target population*: older adult and people with dementia, with preference for those live in long-term settings	NR
	Liu et al., 2015 (USA) [8]	Effectiveness of interventions on eating performance *Target population*: older adults with dementia in LTC	SR
	Abdelhamid et al., 2016 (UK) [12]	Effectiveness of direct interventions on food and fluid intake *Target population*: older adults with dementia or with mild cognitive impairment in any setting	SR + MA
	Bunn et al., 2016 (UK) [32]	Effectiveness of direct interventions on food and fluid intake *Target population*: older adults with dementia or with mild cognitive impairment in any setting	SR
Concept analysis	Chang & Roberts, 2008 (USA) [29]	Characteristics of eating difficulty, its antecedents and consequences providing direction for assessment and management *Target population*: older adults with dementia in any setting	CA on SR
	Aselage & Amella, 2010 (USA) [33]	Characteristics, antecedents and consequences of mealtime difficulties providing direction for assessment and management *Target population*: older adults with dementia	CA

^a^Prevailing aim of the review; *CA* concept analysis; *LTC* long term care; *NH* nursing home; *NR* narrative review; *MA* meta-analysis; *O* overview; *ONS* oral nutritional supplements; *SR* systematic review; *UK* United Kingdom; *USA* United States of America

Watson [6] performed the first overview in the field with the aim of describing the available knowledge on the changes direct and indirect interventions produce on eating difficulties, and a consequent research agenda able to fill the gaps in the evidence available. In 1998, Amella published clinical guidelines based on a review of the diagnosis and management of eating difficulties among older individuals, not specifically those diagnosed with dementia [23]. However, the author dedicated a section of the paper on assessment and management issues regarding individuals with cognitive problems and eating.

Manthorpe and Watson published a third review [25] in the specific field of eating difficulties in dementia care, that summarized the relation between food and dementia—as well as those areas with limited evidence available in need for further investigation. Some years later, Watson and Green [26] published the first systematic review in the field that included studies published from 1993 to 2003: only 13 primary studies that evaluated interventions’ effectiveness were found and just one was a randomized controlled trial.

Aselage et al. [7] conducted a subsequent review that included a purposeful sample of the scientific literature, regulatory and clinical practice guidelines retrieved up to 2010. A total of 28 sources was identified. In the same year, Hanson et al. [28] conducted a systematic review of 25 studies published from 1990 to 2009. Later, Chang and Roberts [27] published an overview that presented multidisciplinary strategies available to help residents with dementia with eating difficulties.

In the past 9 years, eight reviews have summarized interventions’ effectiveness on eating difficulties and mostly involved systematic searches [8, 10, 12, 21, 29–32]. These reviews included studies published in different timeframes: Jackson et al. [29] and Liu et al. [10] included primary studies published from 1999 to 2012 (*n* = 11 studies and *n* = 22, respectively). Liu et al. [8] included studies from 1980 to 2014 (*n* = 11), while Abbot et al. [21] up to 2012 (*n* = 37). More recent reviews included primary studies up to 2013 (*n* = 43 in Abdelhamid et al. [12]), up to 2014 (*n* = 51 in Bunn et al. [32]) and up to 2015 (*n* = 30 in Douglas & Lawrence [31]), respectively. On the other hand, interventions’ effectiveness in increasing fluid intake and/or reducing dehydration risks were reviewed by Bunn et al. [30] including 23 studies up to 2013.

Overall, the findings documented in these reviews showed that many primary studies have been performed to document the effectiveness of interventions; however, these are characterized by a moderate methodological quality; thus, in all reviews included in this scoping review, the need for further research is strongly advised.

Alongside the reviews of empirical studies, in the past decade, two concept analysis have been performed. Chang and Roberts [34] performed the first concept analysis and reported that eating difficulties are due not only to memory and cognitive impairments, but also to several contingent factors that have a probabilistic relation with these difficulties attributable to time or space patterns. Among these, social and psychological factors, as well as the dining environment, and culturally appropriate food choices have been identified.

Aselage and Amella [33] later described the concept of “mealtime difficulties”. They have defined their antecedents (social considerations, cultural factors, lifelong eating patterns), attributes (mealtime patterns and environment, individual and caregiver interactions, dementia, aversive behaviours), and consequences (individual and caregiver stress, compromised nutritional status, loss of eating ability, tube versus hand-to-hand eating, and death). In this concept analysis, the relevance of the resident and caregiver’s interactions was first introduced. Moreover, authors also included the environmental, sociocultural, and contextual implications, thus modifying the research approaches in the field substantially.

### Conceptual frameworks

Only two reviews to date [10, 29] have reported the conceptual framework on which the interventions were based (Constructive Learning Theory; Need-Driven Behaviour Model, Erikson theory), and suggested that there still is a lack of explicit theory-based interventions to improve eating independence among older individuals with dementia. As shown in Table 2, the interventions tested have been based on different underlying conceptual frameworks not reported explicitly in the reviews, ranging from merely biological to more complex processes involving individual, interpersonal, and environmental processes.

**Table 2:** Conceptual frameworks and examples of available intervention studies on feeding difficulties

Conceptual framework	Research examples reported in the included reviews
*Biological processes*	
Structural and transient impairment; Exceed disability [35]	Less supportive environments are significantly associated with eating excess disabilities [8] Enhancing table contrast; visual stimulation during evening meals; high and low visual contrast crockery may reduce transient impairments [21]
Swallowing impairments [36]	Offering appropriate or modified food texture; dysphagia diet food modification [12]
*Cognitive processes*	
Mirror neurons [37]	Sharing meals with staff [12, 32] Encouraging older adult to eat in the dining room to increase intake [29]
Montessori method [38]	Using Montessori-based activities, simplifying the process of mealtime [10] Offering finger food in usual menu [12, 25, 31]
Spaced Retrieval [39]	Recalling the actions required to eat by gradually increasing the delay between each correct recall [8, 10]
Errorless learning model of everyday tasks [40]	Offering verbal prompts, cues, positive reinforcement [7, 8, 26]
*Emotional and behavioural processes*	
Need-driven dementia compromised behaviour (wandering, vocalising, physical aggression) [41, 42]	Offering over lunchtime preferred; ‘quiet’; ‘relaxing’ music; at dinner time, offering music; ‘therapeutic recreation’ music [25, 31] Reducing noise (e.g. from television) and encouraging personal conversation between patient and caregiver; avoiding distractions [31]
Progressively lowered stress threshold [43]	Assessing perceptions: when the staff perceive the patient as combative or uncooperative, less assistance is given during mealtimes [7, 27]
*Emotional and social habits processes*	
Family-style eating [44]	Assessing preferences in terms of breaking meals (or not) with snacks; meal timing, social involvement of caregivers; seasonal variations [7, 30] Creating a family environment; a familiar activity prior to lunch; using standard dinnerware instead of disposable tableware and bibs; table-appropriate height versus eating in wheelchair or in bed [8, 31] Decentralising bulk service as opposed to pre-plated meals; maintaining the ability to serve own food (not-plated) [31]
Familiarity [45]	
*Individual, interpersonal and environment processes*	
Caring [25]	Where individuals with varying levels of dementia ate together without the staff, the person with lower dementia became the caregiver to those with severe dementia [7] Individualising feeding assistance one-to-one; activating the primary nurse in mealtime care; the same carer feeding the patient; enhancing the quality of the interaction between the dyad; offering touch, guidance, redirection, providing compassionate care; offering mealtime assistance [7, 8, 10, 27, 32] Reducing the separation of eating from meal preparation especially for older woman; engaging in meal creation that may stimulate the appetite; food prepared in areas adjacent to or in dining area to stimulate appetite [21, 23, 25, 27] Enhancing dining programmes at NH level; incorporating nutrition as part of good quality care; training staff; offering feeding skills training programmes [10, 21] Changing food service and routines, offering feeding assistance; a training programme on dementia care including supervision sessions and work groups and an environmental redesign; assessing the entire process (e.g. nutritional supplements, changes in food provision) and training carers [31, 32]
Feeding difficulties [34]	
Mealtime difficulties [33]	
Socio-ecological model [46, 47]	
Mealtimes as active processes [48]	
Five Aspects of Meal Model [49]	
Making the Most of Mealtime [50]	

*NH* Nursing Home

### Intervention(s)

As reported in Table 3, reviews summarized available evidence by using different categorizations of interventions. While Watson [6] divided them into direct and indirect interventions, different categorizations have emerged (e.g., training programmes) in the most recent systematic reviews [8, 10, 12, 21, 28–30, 32]. By categorizing these interventions according to Herke et al. [22], the majority of reviews has documented the effectiveness of interventions designed to evaluate environmental modifications, followed by those designed to test the effectiveness of educating family and/or HCPs caregivers.

**Table 3:** Interventions tested according to their classification

		Environmental interventions^**a**^				Behavioural interventions^**b**^		
Author(s), year	Authors’ classifications of interventions	Change of routine	Change of social context	Change of environment	Other^c^	Education/ training of individuals with dementia	Education or training of caregivers	Other^c^
Watson, 1993 [6]	1. Perspective (feeding problems; directing nursing intervention), 2. Research problems (mouthful; individualized changes), 3. Research into feeding problems (index of independence; ethical issues)	*		*	*			
Amella, 1998 [24]	1. History and intake assessment, 2. Intake, 3. Cognition, 4. Environment/ambiance, 4. Relationship with caregiver at meal	*		*			*	
Manthorpe & Watson, 2003 [25]	No classification	*	*	*	*		*	
Watson & Green, 2006 [26]	No classification	*	*	*				
Aselage et al., 2011 [7]	1. Factors influencing mealtime difficulties, 2. Interventions to improve mealtime difficulties	*					*	
Chang & Roberts, 2011 [27]	1. Initiating feeding, 2. Maintaining attention, 3. Getting food into mouth, 4. Chewing food, 5. Swallowing food	*	*	*			*	
Hanson et al., 2011 [28]	1. Studies of high calorie supplements for dementia, 2. Studies of assisted feeding and other intervention	*		*	*			*
Jackson et al., 2011 [29]	1. Educational interventions, 2. Changes to the dining environment and table setting, 3. Changes to menu provision and food service, 4. Increased dietetic input and enhanced nutritional screening	*	*	*			*	
Abbott et al., 2013 [21]	1. Food improvement interventions, 2. Food service, 3. Dining environment, 4. Staff training, 5. Feeding assistance (feeding assistance & food service)	*		*			*	
Liu et al., 2014 [10]	1. Nutritional supplements, 2. Training/education programs, 3. Environment/routine modification, 4. Feeding assistance, 5. Mixed interventions	*		*	*	*	*	
Bunn et al., 2015 [30]	1. Drinking vessel characteristics, 2. Drink characteristics, 3. Physical and social setting for drinking, 4. Institutional factors, 5. Resident assessment instrument minimum data set, 6. Staffing, 7. Ownership and type of facility, 8. Size and location of facility, 9. Care aimed at increasing fluid intake, 10. Care aiming to increase fluid intake and including assistance with toileting	*	*	*	*	*	*	
Douglas & Lawrence, 2015 [31]	1. Feeding assistance, 2. Volunteers, 3. Assistance and training programs, 4. Meal service delivery style, 5. Bulk and buffet-style dining, 6. Family-style dining, 7. Dining room environment and ambiance, 8. Lighting and contrast, 9. Music, 10. Other environment-related considerations	*	*	*	*			
Liu et al., 2015 [8]	1. Interventions to optimize eating performance, 2. Training programs for residents or nursing assistants, 3. Mealtime assistance from nursing caregiver, 4. Environment modification addressing environmental factors, 5. Multi-component interventions addressing personal and environmental factors	*		*	*	*	*	
Abdelhamid et al., 2016 [12]	1. Oral Nutrition supplement, 2. Effects of interventions for swallowing problems, 3. Effects of food and drink modification, 4. Effects of eating and drinking assistance, 5. Effects of interventions with a strong social element around eating and drinking, 7. Finger food, 8. Other food modification, 9. Food modification as part of multi-component intervention, 10. Effects of eating and drinking assistance	*	*	*				
Bunn et al., 2016 [32]	1. Dining environment and food, 2. Education/training, 3. Behavioural interventions, 4. Exercise interventions, 5. Multi-component interventions		*	*	*	*	*	

^a^ According to Herke et al. [22] the environmental modifications cover all changes to the physical surroundings, social context and timing of meals; ^b^ According to Herke et al. [22] behavioural changes cover all changes to knowledge, skill, attitude or habits pertaining to the nutrition of either the person with dementia or those in their immediate vicinity during mealtimes; ^c^ According to Bunn et al. [30] ‘other’ covers interventions where different components are integrated and measured in the same study

Indirect interventions that affect environmental and sociocultural factors have been evaluated since Elmståhl and colleagues’ [51] study. Reducing interruptions and creating family-style meals and buffets, or restaurant- and buffet- style food services have been evaluated and found to be effective in increasing nutritional intake (e.g., Douglas & Lawrence [31]). Dining room redecorations, new furniture, coloured tableware, music, food aromas, and new foodservice also have been found to enhance the residents’ meal experience (e.g., Bunn et al. [32]). Cultural norm expectations, such as appropriate behaviour at mealtimes and the frequency of family visits also have been considered as factors that influence nutritional status [7, 8].

Moreover, observing residents in their dining environment briefly and informally while they are eating or assisted in eating has been documented to improve nutritional status [32]. Financial incentives, organizational culture, and supportive environments also have been documented as factors that may affect eating difficulties and influence the way they are managed [7, 8].

Among the direct or behavioural interventions, studies have been performed with two different targets. Initially the purpose was mainly to increase caregivers’ knowledge, attitudes, and behaviours in mealtime care (e.g., Liu et al. [8]; Amella [24]; Bunn et al. [32]; Douglas & Lawrence [31]). More recently, the aim was to reinstate the individual’s residual abilities through training [8, 10, 30]: an example is the Montessori method that involves breaking down the activity (e.g., eating) into small steps and inviting the individual to complete the tasks him/herself [52].

According to the findings, in the last decade of research, studies have moved from simple, direct interventions, such as hand-under-hand eating [25], to more complex interventions, as Montessori-based activities or specific education training programs for formal and informal caregivers [8, 32].

### Outcomes

Three types of outcomes have been documented in the reviews available, as follows:

#### Eating performance

Improvements in independent eating as measured with the Edinburgh Feeding Evaluation in Dementia tool (EdFED) or with other general or specific tools, have been considered as interventions’ directly affecting the resident (e.g., Aselage et al. [7]). Eating time and the amount of time staff dedicate to assist residents also have been considered an indirect measure of performance (e.g., Liu et al. [8]).

#### Clinical outcomes

Several studies have considered monitoring weight on a monthly basis [32] (e.g., Liu et al. [8]; Bunn et al. [32]). However, given that weight loss may precede clinically dementia by several years, ambiguity in the association between food intake and weight loss and whether or not weight loss precedes the onset of dementia, or the opposite, still remains [25].

According to Amella [24], weighing the person and recording all food consumed are the most accurate intake measures. As a consequence, a 72-h food intake evaluation (including one weekend) in which the number of calories consumed is recorded has been reported as a routine practice in NHs [24]. However, slightly different measurements have been documented as: the total amount consumed or the percentage of the meal consumed; the number of calories; the amount of protein and macro-nutrient intake; and the oral nutrition supplements consumed (e.g. Abbott et al. [21]; Abdelhamid et al. [12]; Bunn et al. [32]; Douglas & Lawrence [31]; Jackson et al. [29]). Some researchers also measured liquid intake or eating and drinking frequency.

Staff may overestimate total intake and fail to identify residents who consume less than 75% of most meals [30]. At the moment, no standardized tool to measure intake is available on which there is international consensus, and thus, the effectiveness of many interventions is unproven [30].

The Body Mass Index (BMI) has also been used as a gold standard to measure the effectiveness of interventions: however, older individuals are subjected to height changes [24] and when they are confined in bed or wheelchairs, different strategies to measure their height should be used (e.g., Chumlea and Guo formula [53, 54]). Researchers have also considered other parameters (e.g., serum albumin, B12) but low levels may suggest an underlying clinical issue [24] suggesting that their specific validity in the context of older individual with dementia should be evaluated.

Finally, the Mini Nutritional Assessment (MNA) is a validated tool [55] that provides a single, rapid assessment of the nutritional status of the elderly patients living in NHs. Its purpose is to evaluate the risk of malnutrition and to evaluate eating interventions’ effectiveness (e.g., Bunn et al. [30]). The MNA categorizes the older individuals in “well nourished,” “at risk of malnutrition,” or “malnourished”: in some studies, this measure has been reported as being closely associated with clinical assessment, albumin, BMI, energy intake, and vitamin status [32].

#### Adverse events or negative outcomes

In the context of older individuals with dementia, adverse outcomes may include aspiration, pneumonia, as well as the psychological burdens associated with the intervention’s intensity as for example wandering, lethargy or agitation [6]. However, few studies to date have measured the above-mentioned negatives which also may result in increased stress among caregivers as in the case of agitation [29].

## Discussion

Since 1977, when the first attempts to measure the effect of a continuous, immediate reinforcement programme on older NH residents’ eating performance were performed [56], researchers’ interest in the topic has increased over the years. A total of 15 reviews has been performed to date that largely targeted research agendas first and adopted systematic review approaches. Among them, only one review focused on fluid intake [32], while the remainder focused on interventions able to promote eating independence. In addition, two concept analyses have been published in the field that have highlighted eating difficulties’ antecedents and consequences and thus are useful to guide future research as well as daily clinical practice.

The target population in the majority of reviews was older individuals with dementia. It would be advisable to report the severity of dementia in future primary and secondary studies, even if this does not necessarily provide information about the degree of eating difficulties, which must be established using validated instruments [6, 7, 57]. Furthermore, the setting has not always been specified and, when reported, individuals living in NHs or long-term facilities predominated. Interventions depend on the context in which they are tested because of environmental factors (at home vs. in a facility). Therefore, there is the need to differentiate interventions’ effectiveness on this basis in future reviews to provide caregivers with evidence appropriate for each care setting.

From the researchers’ perspective, a conceptual framework is a prerequisite to guide researchers in the selection of intervention components that are consistent with studies’ hypotheses [19]. From the HCPs’ perspective, the conceptual framework is also crucial. It helps in understanding the intervention’s rational basis to provide a credible motivation for family caregivers in training processes and also in teaching future HCPs. However, despite general agreement on its importance, conceptual frameworks were found in only two reviews [10, 29] and two concept analyses [33, 34]. The absence of an explicit conceptual framework results in a poor rationale for a causal relation between the intervention and the outcomes desired. Moreover, a weak conceptual framework can delay the identification of effective interventions or the development of cumulative knowledge in a specific field. Nevertheless, in accordance with the inductive process undertaken in this scoping review, results reveal that several implicit conceptual frameworks seem to have been adopted to date in primary studies available. Specifically, some are based on a single process (e.g., biological); others on interrelated processes (e.g., emotional and behavioural social processes, respectively), while others on more complex processes based on individual, interpersonal, and environmental processes. In general, analysed data suggested that there has been a widespread tendency in recent years to test interventions based on more complex processes.

To date, no consensus on interventions’ categorization has been established in this field; however, two different tendencies have emerged. While early authors categorized the interventions tested as direct and indirect [6], in recent reviews different categorizations have emerged, reporting an interest in environment/routine modifications [10], dining environment modifications [21], enhanced dining programs [28], or multi-component interventions [12].

The majority of reviews reported the effectiveness of mealtime routine and/or the dining environment changes. Only a limited number of reviews reported the effects of interventions that change the social context or those at the individual level that offer retraining to improve eating independence. However, the process that explains environmental changes’ effects on eating remains investigated and reported infrequently [32, 58].

In contrast, intervention studies have highlighted the importance of social changes as associated with mealtime factors. For example, eating with others represents a daily pleasure in which the dining room is a place where social and physical domains are interconnected and condition the residents’ mealtime experiences [58]. Despite its relevance, only a few reviews investigated what happens to residents with dementia when the social context changes. Moreover, the findings available are derived from studies with methodological issues [12].

Finally, studies based on behavioural modifications intended to increase the skills of caregivers who provide care during mealtime [33] have been performed suggesting the need for better training in this complex care activity. On the other hand, studies that focus on identifying interventions to re-train individuals with dementia to eat independently are extremely rare [8, 10].

Several outcomes have been measured to date and no consensus has emerged on which end points to use in order to detect the effects of the interventions under study. An intervention’s intensity and duration can influence the degree of eating independence, as well as the clinical (e.g., increased intake) and adverse outcomes (e.g., residents’ agitation). While the first and second outcomes have been reported in the reviews, the third usually has been rarely reported, possibly because the primary studies did not report such endpoints. However, given that each intervention should be evaluated also for its adverse events, more attention in future studies in collecting also negative outcomes is recommended.

### Limitations

This scoping review has several limitations. The only reviews and concept analysis included were those that emerged in the database with the keywords used and published in English. Although two reviewers conducted the search, studies could have been missed, for example those written in other languages or those not indexed as reviews or concept analyses.

The reviews included were conducted at different times and with different degrees of systematization in methods and findings reporting (from overviews to systematic reviews). As a consequence, they can have different degrees of biases in the inclusion of the study as well as in the systematization of the findings.

The categorization performed to identify conceptual frameworks not reported explicitly in the reviews was based on an inductive process followed by content analysis [20]. The educational, professional and research background of the researchers involved may have influenced the findings. Moreover, the examples reported to validate the categorization emerged were not screened for their level of evidence, which limits their utility for clinical purposes.

Finally, according to Arksey and O’Malley [13], no quality assessment of the methodology used in the reviews included or stakeholders’ involvement (clinicians, patients, families, and policymakers) intended to draw insights from the finding has been performed. Moreover, no discussion regarding the funding for the included studies has been performed as required by the PRISMA-ScR guidelines [18] given that all sources were reviews.

### Research and clinical implications

The large number of reviews on intervention effectiveness including several primary studies confirms that research in this field has increased over the years. From the researchers’ perspective, there is the need to summarize the evidence available periodically to support HCPs’ clinical decisions. From the clinical point of view, it is necessary to continue updating competences by accessing the summaries of the evidence produced in the field.

However, given the environment’s influence on eating dependence, to increase the implementation of evidence at the bedside, it is advisable that future studies report the setting where the interventions have been tested in their effectiveness (e.g., at home, NH). More efforts are needed to describe the conceptual frameworks upon which interventions are based, tested, and categorized, as well as the reasons for which they are effective or not. By making the rational basis explicit, HCPs can better inform their decision-making processes, change their practices, as well as they can be more effective in teaching both family caregivers and HCPs.

With the increased understanding of the complexity in the research field of eating dependence among older people with dementia [33], interventions have also improved their complexity by combining different elements. Therefore, future nursing intervention studies should combine direct and indirect interventions at multiple levels (individual, unit, and NH) consistently with the understanding that eating is a complex experience [30, 32]. Future studies should also ensure that the care provided in the context where the research is undertaken is optimal. When poor or sub-optimal care is offered, nursing intervention studies measure the quality improvement’s effects (from poor to optimal care) rather than the intervention’s actual effects. Therefore, it is crucial to measure the care’s baseline quality by involving HCPs who work in the context. It is also advisable to document what HCPs mean by “usual care” in a specific context and provide concrete examples.

A consensus on which outcomes should be measured to achieve homogeneity across studies and thus increase the likelihood of conducting meta-analyses in the field is strongly recommended. In addition to eating performance and clinical outcomes, collecting data on adverse outcomes is also important. Individual acceptance in the short- and long- term on the part of the resident and caregiver should be evaluated as well. At the NHs level, interventions’ long-term feasibility and their costs should be considered at the same time.

With the aim of identifying innovative research questions and of developing well-designed intervention studies, an effective involvement of the HCPs, family caregivers and older individuals with dementia can be useful. Consulting expert practitioners who have wide practical knowledge developed from direct experience represents a unique opportunity for researchers. Through their pragmatic and situation-specific knowledge developed through interactive conversation and experience [59], they can help providing new insights and developing novel interventions to expand future research. Moreover, through their involvement, the implementation of the designed interventions on a large scale and in the long term can increase the strength of the evidence produced in the field. Furthermore, involving care givers as well as older individuals with dementia when possible can also provide insights regarding strategies used at the dyad lever (family caregiver and his/her beloved) to promote and maintain eating independence. Exploring their knowledge in practice can also inform future lines of research based on a personalized approach.

## Conclusions

Despite a large number of studies being available in the field on how to promote and maintain eating independence among older individuals with dementia living in NHs, no intervention can be recommended as gold standard to date. For this reason, a scoping review was performed that included only secondary studies, such as reviews and concept analyses, with the aim of mapping the intervention studies’ features and identifying methodological gaps that should be addressed with future research.

Given the cultural and social influences on mealtime, studies designed to inform daily practices in maintaining or promoting eating independence in older individuals with dementia should focus on complex interventions that include social, cultural, and environmental factors. Moreover, explicit theory-based intervention studies are required to ensure methodological rigor. Alongside the study rationale that must be clear, a full explanation regarding the process justifying the intervention effectiveness is recommended.

Furthermore, residents recruited in studies should be evaluated for their dementia’s severity and eating dependence with validated measures, and the setting where the study is performed (e.g. nursing home, vs. home) should be described, as well as the usual care provided. In evaluating the outcomes, increased agreement on the endpoint and validity measures to adopt is required to compare evidence produced and increase the likelihood that systematic reviews and meta-analyses will be performed. Together with more robust studies, the involvement of clinicians, family caregivers and older residents with dementia can be useful, as their knowledge in practice developed from direct experience can help develop innovative interventions to scrutinise with methodologically sound studies.

## Supplementary information

**Additional file 1: Table S1.** Preferred Reporting Items for Systematic reviews and Meta-Analyses extension for Scoping Reviews (PRISMA-ScR) Checklist [18]

## Data Availability

All the data are available from the corresponding author up on a reasonable request.

## Abbreviations

BMI: Body Mass Index
CA: Concept analysis
CINAHL: Cumulative Index of Nursing and Allied Health
HCPs: Healthcare professionals
LTC: Long term care
NH: Nursing home
NR: Narrative review
MA: Meta-analysis
O: Overview
ONS: Oral nutritional supplements
PRISMA-ScR: Preferred Reporting Items for Systematic Reviews and Meta-analyses
SR: Systematic review
UK: United Kingdom
USA: United States of America
